# Visualization of drug target interactions in the contexts of pathways and networks with ReactomeFIViz

**DOI:** 10.12688/f1000research.19592.1

**Published:** 2019-06-20

**Authors:** Aurora S. Blucher, Shannon K. McWeeney, Lincoln Stein, Guanming Wu

**Affiliations:** 1 Knight Cancer Institute, Oregon Health & Science University, Portland, OR, 97239, USA; 2 Division of Bioinformatics and Computational Biology, Department of Medical Informatics and Clinical Epidemiology, Oregon Health & Science University, Portland, OR, 97239, USA; 3 Ontario Institute for Cancer Research, Toronto, ON, M5G 0A3, Canada; 4 Department of Molecular Genetics, University of Toronto, Toronto, ON, M5S 1A1, Canada

**Keywords:** targeted therapy, drug interaction visualization, Reactome, biological pathway, functional interaction network, Boolean network, constrained fuzzy logic modeling, systems pharmacology

## Abstract

The precision medicine paradigm is centered on therapies targeted to particular molecular entities that will elicit an anticipated and controlled therapeutic response. However, genetic alterations in the drug targets themselves or in genes whose products interact with the targets can affect how well a drug actually works for an individual patient. To better understand the effects of targeted therapies in patients, we need software tools capable of simultaneously visualizing patient-specific variations and drug targets in their biological context. This context can be provided using pathways, which are process-oriented representations of biological reactions, or biological networks, which represent pathway-spanning interactions among genes, proteins, and other biological entities. To address this need, we have recently enhanced the Reactome Cytoscape app, ReactomeFIViz, to assist researchers in visualizing and modeling drug and target interactions. ReactomeFIViz integrates drug-target interaction information with high quality manually curated pathways and a genome-wide human functional interaction network. Both the pathways and the functional interaction network are provided by Reactome, the most comprehensive open source biological pathway knowledgebase. We describe several examples demonstrating the application of these new features to the visualization of drugs in the contexts of pathways and networks. Complementing previous features in ReactomeFIViz, these new features enable researchers to ask focused questions about targeted therapies, such as drug sensitivity for patients with different mutation profiles, using a pathway or network perspective.

## Introduction

The overarching aim of precision medicine is to provide patients with the best choice of therapy based on their individual genetic and/or epigenetic alterations. For cancer and other complex diseases, a key strategy often used is to harness these alterations to stratify patients into subgroups to receive the most appropriate targeted therapy. However, we currently face many challenges such as identifying rigorous biomarkers to guide treatment decisions, rationally designing combination therapies for improved efficacy, reducing therapeutic toxicity and side effects, and managing both intrinsic and adaptive drug resistance to therapy ^1– 3^.

Pathway and network analysis approaches to understanding the interactions between genetic variation and therapeutic response offer researchers the unique ability to understand these interactions in their rich biological context. While pathways are groups of biological entities that are connected together to carry out specific functions or biological processes, networks of entities are usually constructed in a systems-manner, covering many pathways at the same time. In cancer, shared oncogenic pathways in patients may provide an opportunity to stratify and identify therapeutic options. For instance, targeting kinases in the MAPK and PI3K/AKT signaling pathways has been very successful for cancer treatment due to the high prevalence of patient mutations in these pathways ^3, 4^. Note that patients may share the same pathway-level dysregulation that is acquired through alterations in different genes in the pathway or in a set of genes that span multiple pathways. While the first scenario is well-suited to pathway analysis, the second would benefit from network methods that encompass across multiple pathways. Both scenarios provide opportunities for biomarker discovery to inform patient stratification, target prioritization, or treatment assignment by applying pathway and network approaches ^5^.

Pathway- and network-based approaches have also been successfully used in other aspects of targeted therapy. For example, they have been used successfully in prediction of drug side effects and in explaining critical toxicity issues in failed drugs ^6, 7^. They have also been used to unravel the drug resistance mechanisms. Two notable mechanisms of drug resistance in tumors include gatekeeper mutations in direct drug targets (such as those often found in oncogenic kinases) and mutations in non-drug targets that enable bypass resistance pathways used by tumor cells in response to drug-induced inhibition of pathways ^8^. In the second case, mutations in proteins other than the direct target of a drug can confer resistance by activating downstream and/or parallel pathways ^9^.

In each of these applications, it is imperative to be able to visualize the intended effect of drugs on pathways through its designated targets. Currently available resources that offer such visualization include ConsensusPathDB ^10^ and PharmGKB ^11^. ConsensusPathDB aggregates functional interactions including protein interactions, gene-gene interactions, metabolic network interactions, and drug-target interactions from public resources ^10^. With respect to drug-target interactions, ConsensusPathDB allows for selection of interactions for visualization but does not provide functionality for overlaying drugs to a pathway of interest. PharmGKB provides a curated drug-centric view of pathways that aggregates information for a particular drug’s targeted pathways, according to the disease for which the drug is designed ^11^. However, these resources do not provide a means of selecting and filtering drug-target interaction evidence and do not overlay drug-target information to all pathways.

In addition to visualizing both primary and secondary or “off” targets of drugs in the contexts of pathways and networks, we also need to be able to investigate the effect of perturbation either via one drug or a combination of drugs. For this purpose, we need to perform pathway mathematical modeling. In recent decades, several pathway modeling approaches have been developed, including models leveraging ordinary differential equations (ODEs) ^12^, Petri-Nets ^13^, flux-based analysis (FBA) ^14^, probabilistic graphical models (PGMs) ^15^, and Boolean Networks ^16, 17^. ODE modeling is the most sophisticated because it is able to generate both quantitative and dynamic behaviors of model entities. However, lack of initial concentrations and key parameters often limits the application of this approach to small pathways and networks. While Petri-Net and FBA approaches have been used in metabolic network modeling, the sensitivity of these methods to the underlying network structures makes it difficult to apply them to signal transduction pathways because of the incompleteness in many signaling networks due to current limited knowledge. PGMs have been used to integrate multiple 'omics data types together for pathway impact analysis ^15^. However, the need to learn model parameters makes this approach difficult, if not impossible, in many applications due to small sample sizes and/or incomplete network structure. Furthermore, PGMs cannot handle dynamic modeling well since inference results from these models reflect "beliefs" for states of model variables and do not involve kinetic behavior as produced by dynamic modeling.

Boolean network modeling is one of most widely used modeling approaches and has been applied to several large-scale network modeling efforts due to its simplicity and high efficiency. Originally introduced by Kauffman ^18^, Boolean networks provide a method of modeling biological pathways and networks using Boolean variables. Each biological entity in a Boolean network can be in either the active (1) or inactive (0) state, which is determined by logic-based rules involving the network’s functional interactions. A two-state-based Boolean network can be extended to a fuzzy logic model through introduction of continuous variables with values between 0 and 1, inclusively, and applied to biological use cases that involve continuous variables ^19^.

**Figure 1.**
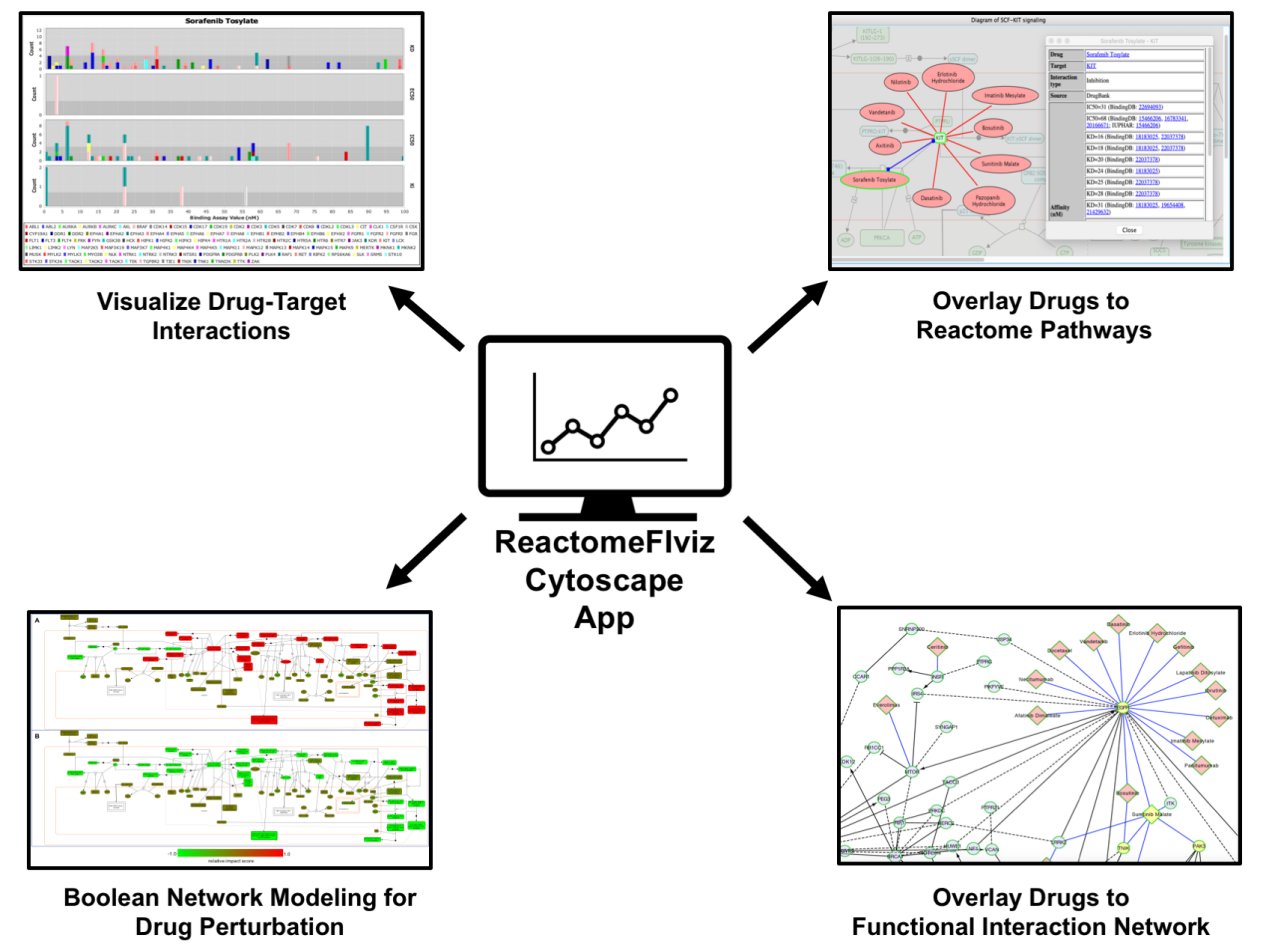
The ReactomeFIviz Cytoscape app enables pathway and network-based approaches in precision targeted therapies. Using ReactomeFIViz, researchers can visualize drug-target interactions according to strength of supporting binding assay evidence, visualize drug-target interactions in the contexts of pathways and functional interaction networks, and perform Boolean Network modeling to investigate the impact of drugs on pathways.

ReactomeFIViz ^20^, a Cytoscape ^21^ app, was developed to perform pathway- and network-based data analysis and visualization for cancer and other complex disease data using Reactome pathways ^22^ and its complementary resource, the Reactome Functional Interaction (FI) network ^23^. Recent enhancements to ReactomeFIViz provide features for visualization of drug-target interactions for approved clinical drugs in the contexts of pathways and networks and modeling the effect of drug action on the pathway activities ( Figure 1). These features offer a versatile and integrative platform to interrogate the full range of drug-target-pathway-network interactions. In this report, we describe these new features and show their utility from different perspectives using a series of practical examples.

## Features with Use Cases

### Visualization of evidence of drug-target interactions

ReactomeFIViz provides features for visualization of drug-target interaction information for 171 FDA-approved cancer drugs from the Cancer Targetome ^24^ and for 2,102 world-wide approved drugs from DrugCentral ^25, 26^, facilitating researchers in investigating supporting evidence for interactions between a drug and all its targets. These features address a major bottleneck in precision therapy research - the lack of rigorous and transparent supporting information on drugs and their targets. While it is well-established that many approved drugs are promiscuous and interact with many targets, it is more difficult to obtain and visualize reliable information about such additional targets ^27, 28^. Resources such as the Cancer Targetome and DrugCentral provide thorough drug-target information, but do not provide visualizations for these drug-target interactions. By adding functionality for researchers to visualize drug-target interactions, ReactomeFIViz empowers investigation of drug-targets in a supporting evidence-based manner.

**Figure 2.**
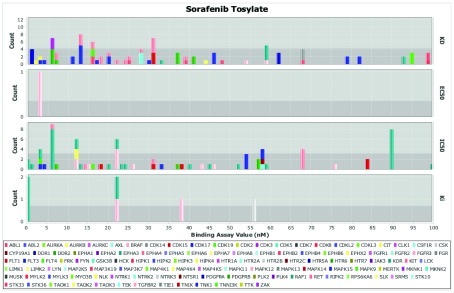
Visualizing drug-target interaction evidence for FDA-approved drug sorafenib via a histogram of drug-target assay values categorized by assay types (KD, EC50, IC50, and Ki). Sorafenib interacts with many targets, even when restricting to target interactions supported by binding assay evidence < 100 nM.

All drugs collected in the Cancer Targetome and DrugCentral can be accessed within ReactomeFIViz. For each drug, target interaction evidence can be filtered according to strength of the supporting assay values and displayed as either a table or as a histogram ( Figure 2). This allows the user to assess drug-target relationships on the basis of supporting evidence. For example, the histogram in Figure 2 for target assay values for the drug sorafenib shows that there are many potential targets with assay values under 100 nM. Targets that have multiple assay values under 100 nM include FLT3, RET, KIT, RAF1, and BRAF. Additional targets with very low assay values include DDR1, DDR2, FLT4, and KDR, among others. Overall there is evidence across multiple assay types to support sorafenib interactions with a variety of targets. Sorafenib is often referred to as a “multi-kinase” inhibitor, and promiscuity (interactions with many targets) has been well noted in the literature ^29, 30^. The ability to display and threshold the full range of supporting evidence is a key requirement for researchers engaged in drug discovery efforts. Some applications, such as the nomination of compounds for drug screens, will require very strong binding assay evidence and would benefit from being able to compare results across multiple assay types. Other applications, especially those that perform *in silico* modeling, may have less stringent requirements for binding assay values. For instance, researchers interested in exploring compounds for drug repurposing may want to look at more weakly binding compounds as a starting place before further optimization.

### Drug-centric perspective on drug-targeted pathways

Depending on the application, different perspectives on drug-target-pathway interaction data are necessary. If we are focused on investigating a particular drug or a small number of related drugs, we would want to explore targets and pathways. For instance, if we want to investigate off-target or toxic effects of a certain drug, we may want to consider all possible targets and pathways with which the drug interacts. In such a scenario, we can look up all the target interactions for a particular drug and map them to pathways. Furthermore, performing enrichment analysis identifies pathways with a significant number of targeted entities, suggesting pathways most perturbed by the drug. The top enriched pathways for sorafenib targets with supporting assay values <= 100 nM are shown in Table 1. Sorafenib is a receptor tyrosine kinase inhibitor, which is known experimentally to interact with a variety of targets. These targets are involved in several signaling pathways. For instance, we can see ( Table 1) that many of sorafenib’s targets are involved in the RAF/MAP kinase cascade as well as other pathways involving VEGF signaling and PIP3/AKT signaling. Examining the full range of pathways targeted by a drug allows us to better understand the drug’s mechanism of action for both efficacy and side effects.

**Table 1.** Top enriched pathways for sorafenib targets with supporting assay values <= 100 nM. Targets for the drug sorafenib with supporting assay values < 100 nM were retrieved from the Cancer Targetome and then mapped to pathways. Pathway enrichment analysis was performed using a binomial test and p-values were FDR-corrected for multiple testing. The table was generated by ReactomeFIViz. Only pathways having FDR <= 0.01 are listed here.

Pathway	Pathway Size	Number of Targets in Pathway	P-value	FDR	Sorafenib Targets in Pathway
Signaling by Receptor Tyrosine Kinases	406	10	1.14E-08	1.07E-06	FLT1,FLT3,FLT4,KDR,PDGFRB,PDGFRA,BRAF, MAPK14,KIT,FGFR3
RAF/MAP kinase cascade	202	8	1.38E-08	1.07E-06	RET,FLT3,PDGFRB,PDGFRA,BRAF,KIT,RAF1, FGFR3
MAPK1/MAPK3 signaling	207	8	1.66E-08	1.07E-06	RET,FLT3,PDGFRB,PDGFRA,BRAF,KIT,RAF1, FGFR3
MAPK family signaling cascades	246	8	6.28E-08	3.01E-06	RET,FLT3,PDGFRB,PDGFRA,BRAF,KIT,RAF1, FGFR3
VEGF ligand-receptor interactions	8	3	1.04E-06	3.33E-05	FLT1,FLT4,KDR
VEGF binds to VEGFR leading to receptor dimerization	8	3	1.04E-06	3.33E-05	FLT1,FLT4,KDR
Neurophilin interactions with VEGF and VEGFR	4	2	4.47E-05	1.21E-03	FLT1,KDR
PI5P, PP2A and IER3 Regulate PI3K/AKT Signaling	90	4	6.10E-05	1.43E-03	PDGFRB,PDGFRA,KIT,FGFR3
Signaling by VEGF	94	4	7.22E-05	1.43E-03	FLT1,FLT4,KDR,MAPK14
Negative regulation of the PI3K/AKT network	95	4	7.52E-05	1.43E-03	PDGFRB,PDGFRA,KIT,FGFR3
Negative feedback regulation of MAPK pathway	6	2	1.00E-04	1.71E-03	BRAF,RAF1
RAF activation	12	2	3.98E-04	6.36E-03	BRAF,RAF1

### Pathway-centric perspective on drug-target interactions

**Figure 3.**
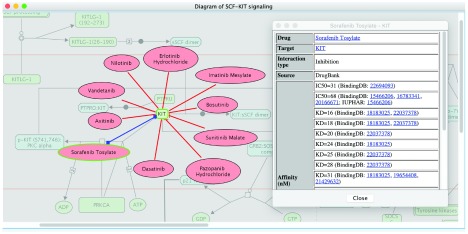
Drugs targeting KIT in the “SCF-KIT Signaling” pathway. Drug-target interactions were fetched from the Cancer Targetome and supported with multiple assay types having values <= 100 nM.

Alternatively, we may start with a pathway of interest and investigate all drugs that target components of the pathway within a selected affinity range. This perspective allows a broad view for assessing how confident we may be able to find a drug targeted to a particular pathway, as many pathways have only weakly or no interacting drugs ^24^. Figure 3 highlights drugs targeting KIT in the pathway “SCF-KIT signaling” ( https://reactome.org/content/detail/R-HSA-1433557), which are retrieved by using the “Fetch Cancer Drugs” feature in ReactomeFIViz to visualize drug-target interactions collected in the Cancer Targetome. As displayed in Figure 3, KIT is targeted by ten different drugs, which are all kinase inhibitors: erlotinib, imatinib, bosutinib, sunitinib, pazopanib, dasatinib, sorafenib, axitinib, vandetanib, and nilotinib ^24, 29^, and supported by assay values less than 100 nM. This view suggests a list of potential drug candidates for researchers to perturb the activity of the SCF-KIT signaling pathway. 

### Constrained fuzzy logic modeling of drug action on pathways

Overlaying drug-target interactions onto a pathway reveals potential drug action targets in the pathway. However, the topological structure of a pathway may be complicated and contain one or more feedback loops. This makes it difficult to predict the dynamic effect of a drug on pathway activity by inspection only. To assist researchers in performing computational modeling of drug-induced pathway perturbation, we developed an automated approach to convert Reactome pathways into Boolean networks (BNs). These BNs were then subject to constrained fuzzy logic simulation ^31, 32^ to model a drug’s perturbation on the activity of a particular pathway. We conducted two constrained fuzzy logic simulations: a reference simulation without considering drug action, and a perturbation simulation considering drug action. To model drug action within the simulation, we mapped drug-target interaction affinity values as “activation” or “inhibition” strength according to the drug’s action mechanism and its assay values collected in the drug data sources. We then used this information to modify the transfer functions related to the drug's target(s) in the fuzzy logic model. Finally, we calculated the relative impact scores for individual entities in the pathway by comparing the model that includes drug effects to the one that does not. For details, see *Methods*.

To illustrate this capability, we take the example of modeling the perturbation induced by the kinase inhibitor sorafenib on the “Signaling by SCF-KIT” pathway. Stem cell factor (SCF), together with its receptor, c-KIT, a tyrosine kinase receptor, regulates pathways related to proliferation, migration, survival, and differentiation of multiple cell types ^33^. These regulated pathways include RAF/MAP kinase signaling, PIP3-activated AKT signaling, and the JAK/STAT signaling pathway. The Reactome-annotated pathway “Signaling by SCF-KIT” includes a set of linked reactions that produce entities activating these regulated pathways.

**Figure 4.**
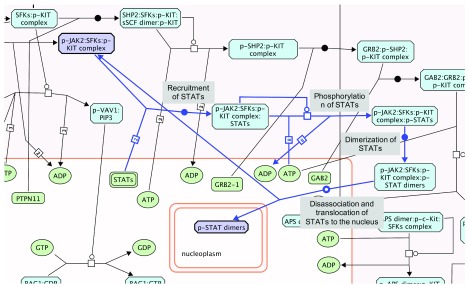
Partial diagram of the “Signaling by SCF-KIT” pathway showing the feedback loop that generates the “p-STAT dimers” complex annotated in Reactome. The entry point of the loop and “p-STAT dimers”, together with four reactions forming the loop, are highlighted in light blue. The reactions are labeled with names in the light-grey boxes. The target of drug sorafenib, KIT protein, is in the upstream of this loop and not shown here.

Drugs targeting proteins involved in SCF-KIT signaling have been under active study for many years ^34^. Evidence shows that sorafenib binds strongly to c-KIT ( Figure 2 and Figure 3) with a minimum binding constant of 16 nM (KD) according to the Cancer Targetome. We performed four constrained fuzzy logic simulations to study the perturbation of sorafenib on the activity of a complex called “p-STAT dimers”, which is one of the major outputs of the SCF-KIT pathway. “p-STAT dimers” activates expression of genes regulated by STAT proteins and is produced by the reaction “Disassociation and translocation of STATs to the nucleus”. This reaction, together with three other reactions, “Recruitment of STATs”, “Phosphorylation of STATs”, and “Dimerization of STATs”, forms a loop, providing a way to produce activated STAT dimer by recycling the “p-JAK2:SFKs:p-KIT" complex ( Figure 4). The four simulations were:

1. Default setup provided by ReactomeFIViz without sorafenib (reference_1 as unperturbed state);
2. Default setup with sorafenib (sorafenib_1 as drug-perturbed state);
3. Default setup with a reduced initial value of PRKCA [cytosol] ( https://reactome.org/PathwayBrowser/#/R-HSA-1433557&SEL=R-HSA-58196&PATH=R-HSA-162582,R-HSA-9006934) from 1.0 to 0.5. No sorafenib was applied (reference_2 as PRKCA-perturbed state). PRKCA is protein kinase C alpha, which phosphorylates KIT and therefore inhibits KIT’s kinase activity ( https://reactome.org/PathwayBrowser/#/R-HSA-1433557);
4. Same as in 3) except that sorafenib was applied (sorafenib_2 as drug/PRKCA-perturbed state).

**Figure 5.**
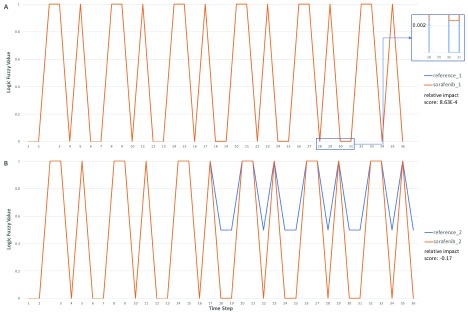
Constrained fuzzy logic simulation results for complex “pSTAT-dimers” in pathway “Signaling by SCF-KIT” from 4 simulations. Panel A: Simulations conducted with the default setup provided by ReactomeFIVIz. Logic fuzzy values for two simulations, reference_1 and sorafenib_1, are almost the same except bottom values starting from step 18: 0.0 for reference_1 and 0.0024 for sorafenib_1 (see the insert for an example). Panel B: Same as in Panel A except the initial value of PRKCA was reduced from 1.0 to 0.5. Logic fuzzy values prior to time step 18 are the same for both reference and sorafenib simulations. The results were exported from ReactomeFIViz and plotted in Microsoft Excel.

**Figure 6.**
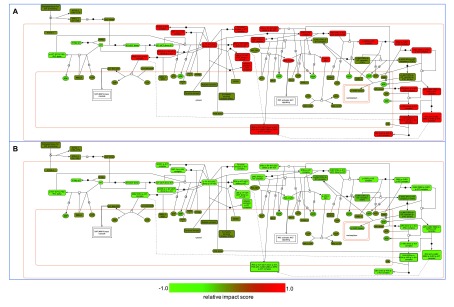
The “Signaling by SCF-KIT” pathway with entities highlighted according to relative impact scores calculated from constrained fuzzy logic simulations. **A**: Relative impact scores for sorafenib with PRKCA’s initial value equal to 1.0 as fully activated. **B**: Same as A except that the initial value of PRKCA was reduced from 1.0 to 0.5.

In Figure 5, we show the activity (labeled as Logic Fuzzy Value in the y-axis) of “p-STAT dimers” over time steps in the simulation. It is well known that pathways having feedback loops usually show periodic cycle patterns or attractors for activities of entities involved in the loops ^35^. As expected, we saw these attractors in all four simulations. In the first drug perturbation modeling (reference_1 and sorafenib_1, Figure 5A) with the initial value of PRKCA equal to 1.0 as fully activated, the application of sorafenib did not notably perturb the activity of “p-STAT dimers” (relative impact score = 8.63E-4), most likely because the loop used to produce “p-STAT dimers” was not affected by sorafenib under this simulation’s condition. However, by reducing the initial value of PRKCA from 1.0 to 0.5, the cycle attractor of “p-STAT dimers” changed from (0, 1, 0, 0, 1, 1) to (0.5, 1, 0.5, 0.5, 1, 1) in the absence of sorafenib (simulation reference_2, Figure 5B), increasing the lower activity in the attractor. Reduction of the initial value of PRKCA brought down the activity of “p-KIT (S741,746):PKC alpha”, the inhibitor of the reaction, “Autophosphorylation of KIT”, resulting in an increase of the activity of “p-JAK2:SFKs:p-KIT complex” via several intermediate reactions. “p-JAK2:SFKs:p-KIT complex” is the entry point of the loop that produces “p-STAT dimers” ( Figure 4). Increasing the activity level of this complex levels up the p-STAT dimers, thereby increasing the lower value of its cycle attractor. Intriguingly, the application of sorafenib abolished the effect of the reduction of PRKCA’s initial value, bringing down the lower value of the cycle attractor from 0.5 to 0.0024, the same value when PRKCA’s initial value was set at 1.0. Hence, sorafenib had a much stronger perturbation when PRKCA’s initial activity was lower (relative impact score = -0.17). For the detailed pathway annotation, see Figure 6 and also refer to the Reactome pathway diagram: https://reactome.org/PathwayBrowser/#/R-HSA-1433557.

The above comparison analysis of the simulation results demonstrates the importance of mathematical modeling, even without experimentally measured parameters, by predicting that alterations in the PRKCA gene may impact the effects of the drug sorafenib on SCF-KIT signaling. For instance, a loss-of-function mutation in the PRKCA gene could influence how sorafenib affects the SCF-KIT pathway. Alternatively, another drug that inhibits PRKCA could be used in combination with sorafenib to potentially impact the pathway in a different manner than with sorafenib alone. This level of mechanistic and dynamic investigation is not possible by merely overlaying drug-target interactions onto the pathway.

We also performed analysis for another output of this pathway, “PI(3,4,5)P3” ( https://reactome.org/PathwayBrowser/#/R-HSA-1433557&SEL=R-ALL-179838) ( *Extended data:* Figure S1), which was annotated as an activator for pathway “PIP3 activates AKT signaling”. We observed similar behavior as in the case of “p-STAT dimers”: reducing the initial value of PRKCA increased the activity of “PI(3,4,5)P3”. The application of sorafenib modestly increased PI(3,4,5)P3's activity using default PKCA activity level (1.0), but reduced its activity when PRKCA’s activity was reduced. However, the relative impact scores for PI(3,4,5)P3 are much larger compared to the case of the p-STAT dimers described above, most likely because this entity has a converged single stable activity, in contrast to p-STAT dimer’s cycle attractor.

We’d like to emphasize that the above simulations were performed using a set of initial values and transfer function parameters that we developed to ensure simulations with converged solutions. In reality, the actual tumor cell conditions will likely differ from the parameters we used. ReactomeFIViz provides a set of intuitive user interfaces for users to try different parameters in simulation. Future work for this modeling framework will include approaches for estimating and learning these parameters based on large scale omics datasets. We hope to address this daunting issue soon.

To assist researchers in investigating drug impact on pathway activities in a systematic way, ReactomeFIViz provides a feature to highlight entities based on relative impact scores. Figure 6 shows SCF-KIT Signaling pathway highlighted according to the relative impact scores with PRKCA’s initial value equal to 1.0 ( Figure 6A) and 0.5 ( Figure 6B) for sorafenib.

### Network perspective on drug-target interactions

A network perspective unites multiple pathways together, allowing a bird’s eye view of drug-target relationships. This perspective is especially important when considering drugs with targets that span multiple pathways or considering potential crosstalk between pathways (e.g. Table 1). Projecting drug-target interactions onto a network helps prioritization of targets, targeted pathways, and targeted therapies. Here, we use the TCGA ovarian cancer data ^36^ as an example to show the utility of this network perspective for prioritizing targeted therapies for ovarian cancer.

The Reactome functional interaction (FI) network was constructed by extracting functional relationships from manually curated pathways in Reactome and several other pathway databases, and then combining them with predicted interactions based on a machine learning approach, Naïve Bayes Classifier ^23^. Using genes mutated in TCGA ovarian cancer samples, we constructed a FI network, where each node represents a gene mutated in at least 5 patients in the TCGA cohort, and each edge represents a functional interaction between two genes.

**Figure 7.**
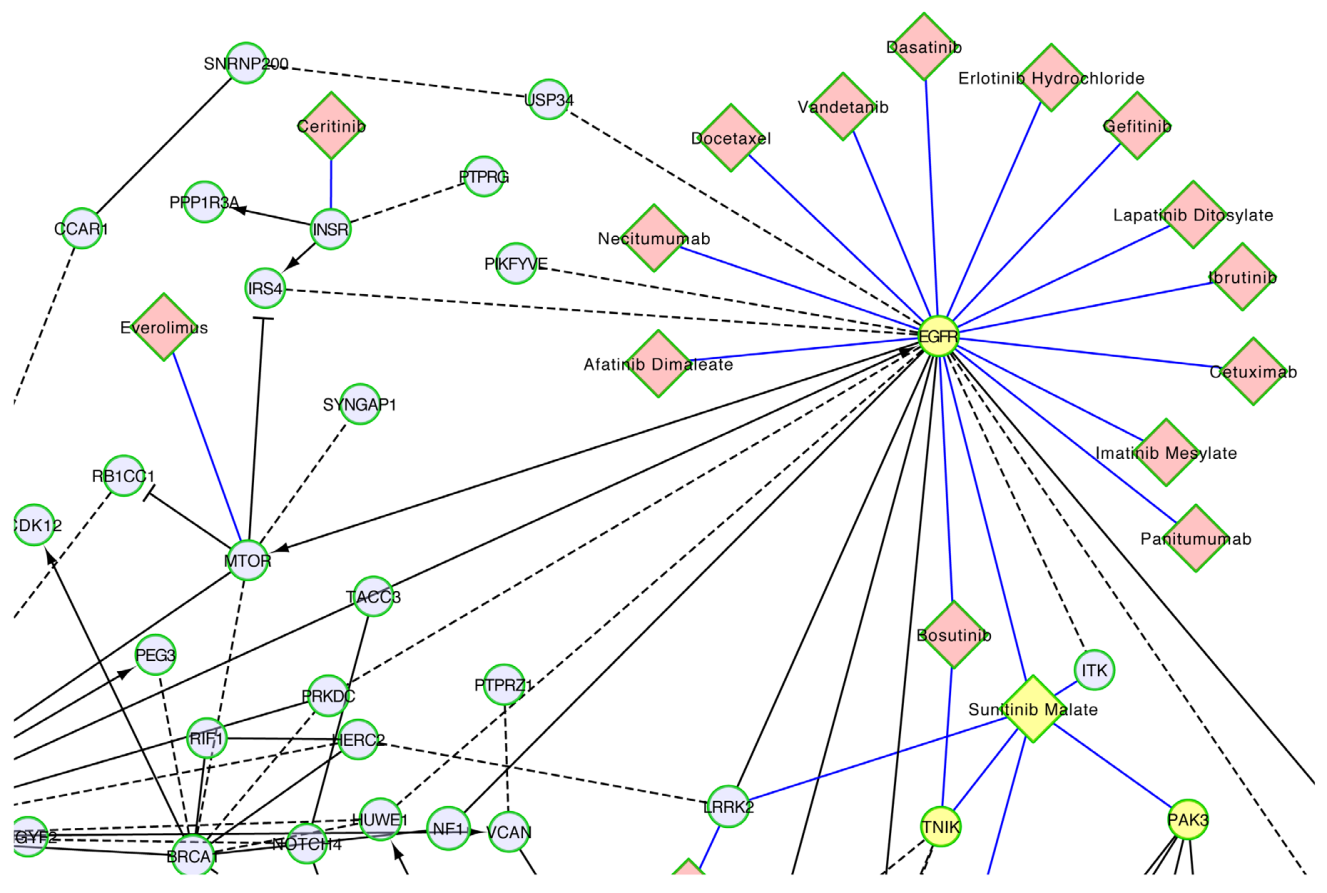
Functional interaction network for frequently mutated genes in TCGA ovarian cancer samples. Only part of the FI network was shown here for drugs targeting protein products of genes in the network. Network nodes are genes mutated in at least 5 TCGA samples, where size of the node indicates the number of samples with the mutated gene. Black edges are FIs between genes, blue edges drug-target interactions with <= 100 nM supporting assay values, dashed edges predicted FIs, solid edges extracted from annotated FIs, -> activation or catalysis FIs, -| inhibition FI, - FIs extracted from complexes or inputs of reactions. EGFR, TNIK, PAK3 and sunitinib malate are highlighted in yellow.

Combining the TCGA ovarian cancer FI network with the Cancer Targetome reveals that there are 18 different cancer drugs in the Cancer Targetome that potentially interact with target proteins in the ovarian cancer set, at affinity values <= 100 nM. One strategy for prioritizing drugs interacting with members of this network would be to prioritize drugs targeting proteins that are also members of multiple significantly enriched pathways for the ovarian cancer cohort. For instance, epidermal growth factor receptor (EGFR) may be prioritized as a target for ovarian cancer. It has roles in multiple pathways that are significantly enriched for mutated genes, including “L1CAM interactions”, “Focal adhesions”, “Calcium signaling pathway”, “PI3K-AKT signaling pathway”, and “HIF-1 signaling pathway” ( *Extended data:* Table S1). EGFR is targeted by both monoclonal antibody drugs and kinase inhibitors ^37^. 14 of the 18 drugs that interact with targets in the network bind to EGFR ( Figure 7). EGFR is a member of the well-studied ERBB protein family and is a target of interest for ovarian cancer ^37^. Many studies have shown encouraging results for the effect of EGFR and other ERBB-family inhibitors on ovarian cancer cell lines ^38^. However, despite promising preclinical evidence, inhibitors targeting the ERBB signaling have up to this point shown little efficacy in patient clinical trials ^38^. Current efforts in this area are focused on the use of ERBB-family inhibitors in combination with cytotoxic or targeted therapies ^39, 40^.

An alternative strategy for prioritizing drugs interacting with this FI network would be to select drugs interacting with targets in multiple, disjoint pathways to potentially achieve a greater coverage of key genes in the network. For instance, the drug sunitinib malate targets both “TRAF2 And NCK Interacting Kinase” (TNIK) and “P21 (RAC1) Activated Kinase 3” (PAK3) ( Figure 7), which are individually involved in different pathways enriched by mutated genes ( *Extended data:* Table S1). 

## Discussion

Pathway and network-based approaches for precision medicine offer a holistic view of drug action, providing biological contextualization for drug-target interactions. We have developed a user-friendly, integrative software platform by enhancing a popular Cytoscape app, ReactomeFIViz, for the community to perform pathway and network-based drug-target interaction visualization, modeling, and analysis.

Reactome ^22^ is one of the most popular biological pathway databases, providing high quality manually curated biological pathways that cover over 50% of known human genes. Pathways in Reactome are annotated according to biochemical reactions, which are connected together to form networks. We developed a scheme to automatically convert reaction-based pathways in Reactome into Boolean networks and then perform constrained fuzzy logic based simulation ^31, 32^. Our approach is generic and can be adopted for other reaction-based pathway databases (e.g. Panther Pathways ^41^) and pathways provided in the BioPAX format ^42^, the community standard for pathway data exchange.

Future improvements to our Boolean network-based fuzzy logic modeling approach will find a better measure to quantify the impact of drug perturbation on entity activities. The current relative impact score may be too sensitive when the area under curve of the fuzzy logic values vs time steps generated from the reference simulation is very close to 0, as shown for the PIP3 example ( *Extended data:* Figure S1). Future improvements will also include expansions for learning and/or optimizing parameters based on omics data and improving handling of entity set members for tissue-specific simulation by annotating tissue-specific information. Furthermore, collaboration with bench scientists will help to validate and refine the computational models.

An unmet need in understanding drug resistance is the need for additional mechanistic information on how mutations in drug targets may affect drug-target binding, one of the major causes of resistance ^8^. The major drug-target interaction data resources, including Cancer Targetome and DrugCentral, have not collected this type of information yet, and ReactomeFIViz cannot perform pathway modeling for this type of ‘on-target’ resistance ^8^. We are working on introducing features to ReactomeFIViz to help users visualize protein mutant locations in protein-protein interaction 3D structures by utilizing the structural data generated by Mechismo, a software tool used to infer 3D structural information and functional impact of structural variants on protein-protein and protein-chemical interactions ^43, 44^. We plan to integrate these features with drug-target interactions once this type of data is available in the future.

A second challenge in understanding drug resistance is pathway-level mechanisms of resistance. Bypassing drug-targeted pathways is another common mechanism for cancer drug resistance ^8, 9^. An improved understanding of pathway redundancy and crosstalk inside cells will help us to uncover the mechanisms driving this type of drug resistance. However, the majority of pathway databases, including Reactome, do not yet provide a systematic pathway and network map for visualization and modeling of pathway crosstalk. We envision an integrative systems pathway map in the future, including signaling transduction, gene regulation, and metabolism, that will aid in this type of analysis.

Overall, visualization of drug-target interactions in the contexts of pathways and networks and *in silico* modeling of drug perturbation allow for prioritization of drugs with greater promise for effective pathway targeting in cancer cells. This is particularly valuable for drug combination discovery, as the number of possible drug combinations for even a modestly sized set of monotherapies can be intractable to test experimentally. Promising leads from perturbation analysis can be prioritized for bench testing. Furthermore, the examples discussed here are applicable for drugs outside the cancer domain. We foresee a routine adoption and use of pathway and network-based prioritization methods for repurposing drugs from one therapeutic domain into other domains ^45^.

## Methods

### Cancer Targetome

The Cancer Targetome ^24^ aggregates drug-target interactions from four public databases: DrugBank ^46^, Therapeutic Targets Database ^47^, the IUPHAR/BPS Guide to Pharmacology ^48^, and BindingDB ^49^. This resource provides interaction information across 171 FDA-approved cancer drugs, 880 protein targets, and over 6800 interactions between these drugs and targets. Each drug interaction is accompanied by supporting evidence across three tiers ^24^: parent database, PubMed IDs for literature references, and experimental assay values. Experimental assay values include IC50, EC50, KD, and Ki values. Each drug-target interaction may be supported by multiple types of assay values. The companion supporting evidence allows for assessment of putative drug-target interactions in an empirical data-driven manner, in particular, for consideration and inclusion of secondary or “off” targets of drugs that may play an important role in a drug’s effects on a particular pathway. For more information, see 24. The version currently supported in ReactomeFIViz was developed in 2016.

### DrugCentral

The DrugCentral ^25^ database collects extensive drug information, including but not limited to compound structure, active ingredients, and mechanism of action for approved drugs from the US Food and Drug Administration (FDA), European Medicines Agency (EMA), and the Japan Pharmaceuticals and Medical Devices Agency (PMDA). Target information in DrugCentral is mined from bioactivity resources such as ChEMBL and IUPHAR, and may also be supplemented with manually curated target information from drug approval labels. Overall, DrugCentral (version released in August, 2017) includes information for 2102 compounds, 1550 targets, and 13,407 drug-target interactions. The drug-target interaction file supported by ReactomeFIViz, drug.target.interaction.08292017.tsv, was downloaded from the DrugCentral web site ( http://drugcentral.org) in 2017.

### TCGA ovarian cancer dataset

For the functional interaction network in Figure 7, we used the Gene/Mutation Set Analysis feature in ReactomeFIViz to create a network with genes mutated in at least 5 samples in the TCGA Ovarian Cancer dataset ^36^. Pathway enrichment p-values in ReactomeFIViz were calculated using the binomial test and were false discovery rate (FDR)-corrected based on the Benjamini-Hochberg approach ^50^.

### Construction of Boolean networks from Reactome pathways

**Figure 8.**
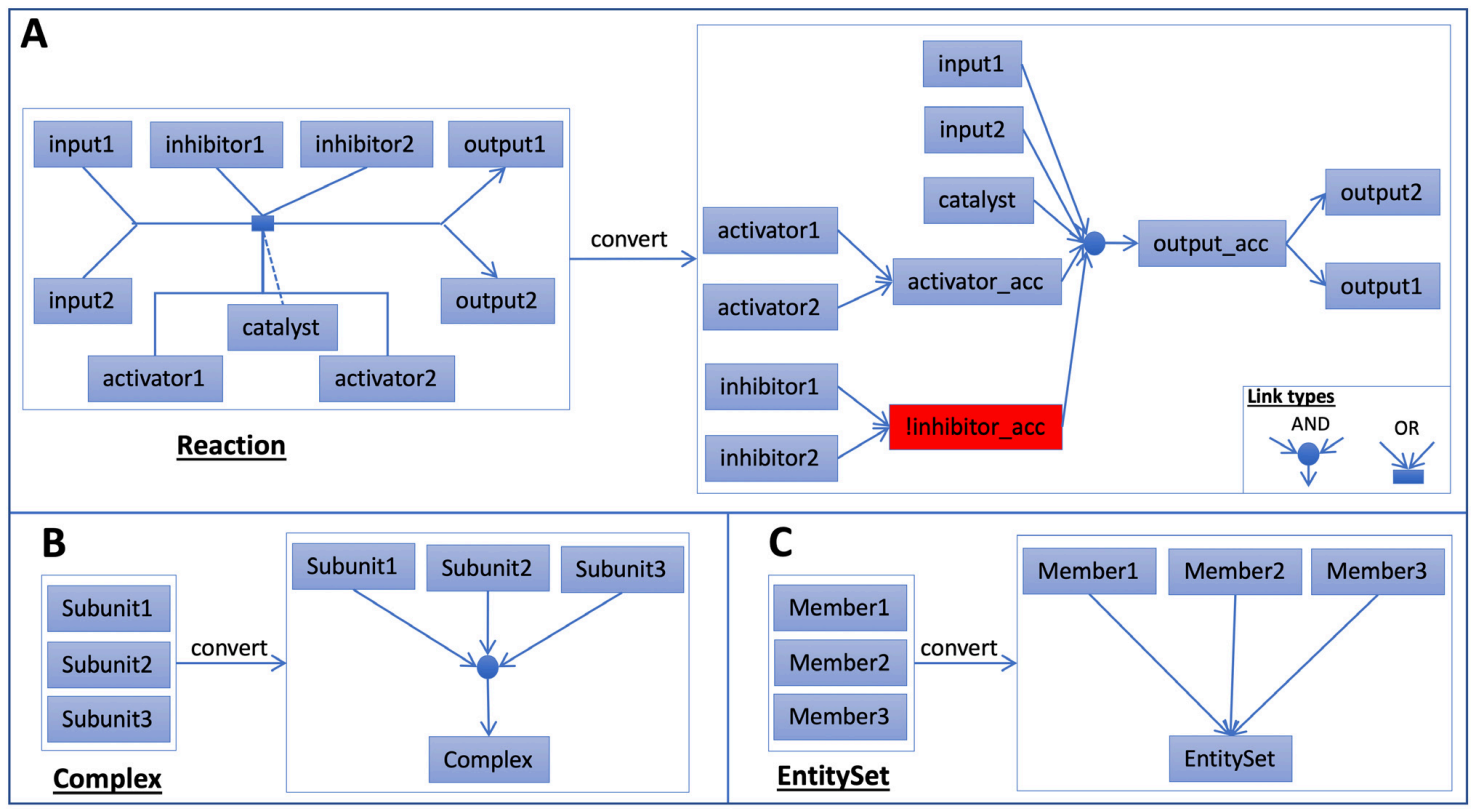
An automatic scheme to convert Reactome pathways into Boolean networks by handling Reaction, Complex and EntitySet instances. **A**: Convert a typical Reactome reaction into a set of Boolean relationships. **B**: Convert a complex into an AND relationship between complex subunits and the complex. **C**: Convert an EntitySet into an OR relationship between set members and the set.

In Reactome, the biochemical reaction is the basic unit for pathway annotation. Pathways are annotated as a set of biochemical reactions that are linked together. We developed a scheme to automatically convert Reactome pathways into Boolean networks ( Figure 8) by adopting our previous approach used to convert Reactome pathways into probabilistic graphical models ^20^ based on the PARADIGM approach ^15^. A typical reaction in Reactome has one or more inputs, one catalyst, one or more activators, one or more inhibitors, and one or more outputs. A logical “AND” relationship was created among inputs, the catalyst, and activators. Inhibitors were negated and then added into this “AND” relationship. To make the relationship simple, if multiple outputs, activators, or inhibitors are annotated for a reaction, we introduced accessory nodes for them. Therefore, a typical reaction in Figure 8A was converted into a set of Boolean relationships (· for AND, + for OR, ! for NOT):

- input1 · input2 · catalyst · activator_acc · !inhibitor_acc = output_acc
- activator1 + activator2 = activator_acc
- inhibitor1 + inhibitor2 = inhibitor_acc
- output_acc = output1
- output_acc = output2

Reactome has annotated many complex assembly reactions. However, some complexes are annotated directly for reactions without explicit description of how subunits are assembled together. We expanded a complex like this into an “AND” relationship among its subunits and generated the following Boolean relationship for the complex as illustrated in Figure 8B:

- Subunit1 · Subunit2 · Subunit3 = Complex

EntitySet is a class in the Reactome data model ^51^ used to collect a set of entities with the same function in a biochemical reaction and is routinely used to collect protein isoforms. We expanded an EntitySet instance into an “OR” relationship among annotated members:

- Member1 + Member2 + Member3 = EntitySet

Thus, by converting biochemical reactions, complexes, and EntitySets into a set of Boolean relationships, we converted a Reactome pathway into a Boolean network.

### Constrained fuzzy logic simulation of drug perturbation on pathways

We adopted the constrained fuzzy logic simulation approach ^31, 32^ to simulate the dynamic behavior of Boolean networks that are automatically converted from Reactome pathways by extending two-state Boolean variables to continuous variables with values between 0 and 1, inclusively. This allows our modeling approach to be applied to continuous ‘omics data types and drug-target assay values without the need for discretization. Based on the minimum supporting assay values for drug-target interactions loaded from the drug data sources, we calculated inhibition or activation strength by using the following hard-coded piecewise function:

$$\left\{ \begin{array}{l} \;0.999\;if\;assay\;value<1\;nM\\ \;0.99\;if\;assay\;value<10\;nM\\ -0.196*(\text{log}(assay\;value)-1)\;+\;0.99if\;assay\;value<10^{6}\;nM\\ \;0 \end{array}\right.$$

Function *f(input)* may be a Hill function ^31, 32^ or an Identity function. For the four simulations conducted with the “Signaling by SCF-KIT” pathway, the Identity function was used.

To measure the perturbation impact caused by drug action, we developed a relative impact score based on the time step plots for individual entities in the pathway. We conducted the simulation twice: one with drugs as the perturbation, and another without drugs as the reference. The relative impact score was then calculated as follows (AUC for area under curve of fuzzy logic variable value vs. time step):

$$relative\_impact\_score=\frac{perturbation\_AUC-reference\_AUC}{perturbation\_AUC+reference\_AUC}$$

Our test results indicated that the above defined relative impact score, ranging between -1.0 and 1.0, inclusively, converged within a limited number of time steps in most cases.

### Software implementation

We adopted the three-tier software architecture: The data tier provides drug-target interactions from the Cancer Targetome and DrugCentral, contents from the Reactome database, and the Reactome FI network; The server tier fetches the content from the data tier and then send it to the front-end app via a RESTful API (application programming interface). The API also provides methods for Boolean network construction and fuzzy logic simulation. The server was implemented upon the Spring framework ( https://spring.io) along with the Hibernate OR (object/relational) mapping ( http://hibernate.org); the front end provides all user interfaces.

Our implementation of Boolean network in Java referred to the Java implementation of open source CellNOptR ^52^, a comprehensive Boolean network modeling toolkit for 'omics data, hosted at https://github.com/saezlab/cytocopter/tree/master. The implementation of constrained fuzzy logic simulation referred to the code (version 1.2) hosted at https://github.com/saezlab/CNORfuzzy by Saez Lab. The simulation ran until either the maximum difference between two consecutive iterations was less than 1.0 × 10 ^-6^, or the total number of iterations reached the larger of 100 or 1.2 × total number of variables in the Boolean network. To calculate the relative impact scores, we expanded the plots of fuzzy variable values vs. time steps based on reached attractors by 20 time steps each time. This expansion stopped when the maximum difference between two consecutive expansions was either less than 0.01, or the expansion reached a total of 1000 time steps. As the default setup for simulation, 1.0 was assigned as the initial values for those entities annotated as inputs of reactions forming loops or inputs that are not annotated as outputs in the pathway.

We used Java 8 ( https://java.com) and Eclipse ( https://www.eclipse.org) as the integrated development environment (IDE) for programming. ReactomeFIViz is released through the Cytoscape App Store ( http://apps.cytoscape.org/apps/reactomefiplugin). The code is open source, hosted at GitHub ( https://github.com/reactome-fi/CytoscapePlugIn).

## Software availability

Home page for user guide describing procedures on how to use ReactomeFIViz features for drug visualization and modeling: https://reactome.org/tools/reactome-fiviz

Cytoscape app store: http://apps.cytoscape.org/apps/reactomefiplugin

Latest source code: https://github.com/reactome-fi/CytoscapePlugIn

Source code as at the time of publication: https://github.com/reactome-fi/CytoscapePlugIn/releases/tag/f1000_drug_paper

Archived source code as at the time of publication: http://doi.org/10.5281/zenodo.3237955 ^53^

License: Creative Commons Attribution 4.0 International (CC BY 4.0) License ( https://reactome.org/license).

## Data availability

### Underlying data

The TCGA ovarian cancer mutation data file: http://cpws.reactome.org/caBigR3WebApp2018/ov.maf.txt.zip

### Extended data

Zenodo: Reactome_FIViz_Drug_Visualization_Supp, https://doi.org/10.5281/zenodo.3239441 ^54^

This project contains the following extended data:

**Table S1.** Significant pathways for the TCGA ovarian cancer FI network. Pathways enriched by the mutated genes in the TCGA ovarian cancer FI network with FDR <= 0.05 are listed. Highlighted pathways contain EGFR (in yellow except Focal adhesion(K)), PAK3 (in brown), or TNIK (in green), as discussed in the main text. (R) indicates pathways collected from Reactome, (K) from KEGG (1), (N) from NCI Pathway Interaction Database (2), (P) from Panther Pathways.

1. Kanehisa M, Furumichi M, Tanabe M, Sato Y, Morishima K. KEGG: new perspectives on genomes, pathways, diseases and drugs. Nucleic Acids Res. 2017 Jan 4;45(D1):D353–61.

2. Schaefer CF, Anthony K, Krupa S, Buchoff J, Day M, Hannay T, *et al.* PID: the Pathway Interaction Database. Nucleic Acids Res. 2009 Jan;37(Database issue):D674–9.

**Figure S1.** Constrained fuzzy logic simulation results for entity PI(3,4,5)P3 [plasma membrane] ( https://reactome.org/PathwayBrowser/#/R-HSA-1433557&SEL=R-ALL-179838&PATH=R-HSA-162582,R-HSA-9006934) in the “Signaling by SCF-KIT” pathway from 4 simulations. A: Simulations conducted with the default setup provided by ReactomeFIVIz. B: Same as A except the initial value of PRKCA was reduced from 1.0 to 0.5. Logic fuzzy values prior to time step 11 are the same for all four simulations, which is 0.0.

Data are available under the terms of the Creative Commons Zero "No rights reserved" data waiver (CC0 1.0 Public domain dedication).
