# Supplementary material for: Visualization of drug target interactions in the contexts of pathways and networks with ReactomeFIViz
Source: F1000Res. 2019 Jun 20;8:908. [Version 1] doi: 10.12688/f1000research.19592.1 (PMC6644836; doi:10.12688/f1000research.19592.1)
Supplement: Peer review history

# Peer review: Reviewer response for version 1

**DOI:** 10.5256/f1000research.21482.r50787

**Published:** 2019-07-26

**Authors:** Tero Aittokallio, Ziaurrehman Tanoli

**Affiliations:** 1 Institute for Molecular Medicine Finland (FIMM), University of Helsinki, Helsinki, Finland; 2 University of Helsinki, Helsinki, Finland

This manuscript introduces an enhanced Cytoscape plugin, called ReactomeFIViz, which enables researchers to visualize drug-target interactions according to strength of supporting binding assay evidence, visualize drug-target interactions in the contexts of pathways and functional interaction networks, and to perform Boolean Network modeling to investigate the impact of drugs on pathways. This seems like a highly useful tool for the community, and I have only a couple of comments on how to improve the manuscript, as well as a few suggestions on potential future versions of ReactomeFIViz.

1. There are several drug-target interaction network visualization and drug repurposing analysis tools available as web-based software implementations, for instance, those reviewed in our recent article ^1^. Please cite those that are relevant for the present work, and also describe the added value of ReactomeFIViz in the context of precision medicine applications.
2. Cancer Targetome aggregates drug-target data from various databases in different formats; for example, DrugBank simply reports binary drug-target interactions and does not provide quantitative bioactivity measurements (i.e. IC50, Kd, Ki). Similarly, IUPHAR/BPS Guide to Pharmacology reports median bioactivity values in negative log. Please describe how these data were combined?
3. It remains unclear how the evidence from multiple bioactivity assays (e.g. Kd, Ki, EC50, IC50) are summarized into an interaction strength for pathway analyses (as shown in Figure 2 and Table 1). This is important especially when there are multiple, perhaps contradicting evidence from multiple assays for the same drug-target pair; which assay is considered most informative in such cases?
4. We have initiated an open-data platform, called DrugTargetCommons (DTC), with aim to annotate bioactivity data with assay information to enable more harmonized compound-target analyses. This community-based effort involves both approved drugs and investigational compounds, and perhaps something the authors would like to consider in the future version of ReactomeFIViz.
5. The drug-target interaction network analyses shown in Fig. 7 are interesting, but it remains unclear whether the extracted drug-target information includes also mutant targets, or is the visualization based only on wild-type targets? Selective targeting of the mutant protein (e.g. oncogene) often leads to better efficacy and less side effects, especially if the protein is essential in healthy cells. For instance, DTC includes more than 200 mutant targets with binding affinities for hundreds of drugs.
6. Introduction mentions several current challenges in precision medicine, such as identifying biomarkers to guide treatment decisions, rationally designing combination therapies for improved efficacy, reducing therapeutic toxicity and side effects, and managing both intrinsic and adaptive drug resistance. The authors should summarize in Discussion which of these challenges ReactomeFIViz can currently address, and how, and which will be topics of future research. 

Specific comments:

- Figure 1: the text is so small in the panels that it becomes unreadable.
- Figure 2: several targets are marked with same colors, can you redefine coloring based on pathways?

We confirm that we have read this submission and believe that we have an appropriate level of expertise to confirm that it is of an acceptable scientific standard.

## References

[1] : Interactive visual analysis of drug-target interaction networks using Drug Target Profiler, with applications to precision medicine and drug repurposing. *Brief Bioinform*.2018; 10.1093/bib/bby119 10.1093/bib/bby119 30566623

# Peer review: Reviewer response for version 1

**DOI:** 10.5256/f1000research.21482.r50435

**Published:** 2019-07-19

**Authors:** Aritro Nath

**Affiliations:** 1 Department of Experimental and Clinical Pharmacology, University of Minnesota, Minneapolis, MN, USA

In this article, Blucher et al. present important updates to the ReactomeFIViz app for Cytoscape. They added several very useful features to this popular plugin that now enables easy visualization of drug and their targets in the context of biological pathways. In addition to retrieving and overlaying the drug-target information on the Reactome pathways, the updated app provides convenient visual representations of several layers of related information. Moreover, they added the capability to model the impact of single or multiple drugs on the pathways using a simple yet elegant approach based on Boolean Network modeling. Overall, the authors have done a great job in providing a thorough description of the new capabilities of the software.

 I only have some minor comments and observations:

1. The fuzzy logic modeling examples are very well described but It might be useful if the description included some discussion on the biological interpretation of these results, especially in the context of precision medicine.
2. I found the online documentation for the reactomeFIViz documentation very useful to follow the analyses discussed in the manuscript. However, I was not able to test the “systems pathway impact analysis” which kept returning an HTTP status 500 error message. I am not sure if it’s a user-specific error, but it might be worth checking.
3. Some of the drug-target overlays are very difficult to read because of high edge-density. It might help if the edges were semi-transparent.  
4. Consider using a color-blind friendly palette for visualizing simulation outputs, as the red-green gradient may be challenging for some users to differentiate.

I confirm that I have read this submission and believe that I have an appropriate level of expertise to confirm that it is of an acceptable scientific standard.

# Peer review: Reviewer response for version 1

**DOI:** 10.5256/f1000research.21482.r50786

**Published:** 2019-07-15

**Authors:** Fuhai Li

**Affiliations:** 1 Institute for Informatics, Washington University School of Medicine (WUSM), Washington University in St. Louis, St. Louis, MO, USA

In this study, the authors improved a cytoscape network tool to analyze and visualize drug target interaction in the context of signaling pathways. The detailed examples are very helpful to indicate the importance of the functions of the tool. Overall it is well written. A couple of comments are as follows:

1.  Discuss the possibility of zoom in/out the signaling pathways, considering that multiple signaling pathways are often activated in a specific disease. If it is possible to list/visualize all drugs targeting on all the pathways, and users can check the details, like the google map.
2. For the drug-target interaction, it is better to indicate the target names directly beside the color bar (because it is hard to match so many colors to drug names in the legend area)
3. Minor: the resolution of Figure 1 is low. The subfigures (though these sub-figures are presented again the manuscript) are not clear. 

I confirm that I have read this submission and believe that I have an appropriate level of expertise to confirm that it is of an acceptable scientific standard.
